# *In Vitro* Phytochemical, Antibacterial, and Antifungal Activities of Leaf, Stem, and Root Extracts of *Adiantum capillus veneris*

**DOI:** 10.1155/2014/269793

**Published:** 2014-01-28

**Authors:** Muhammad Saqib Ishaq, Muhammad Medrar Hussain, Muhammad Siddique Afridi, Ghadir Ali, Mahrukh Khattak, Sohail Ahmad, Shakirullah

**Affiliations:** ^1^Department of Microbiology, Faculty of Life Sciences, Abasyn University, Peshawar 25000, Pakistan; ^2^Medicinal Botanic Centre, PCSIR Labs Complex, Peshawar 25120, Pakistan; ^3^Department of Microbiology, Shaheed Benazir Bhutto Women University, Peshawar 25000, Pakistan; ^4^Department of Chemistry, Kohat University of Science and Technology, Kohat 26000, Pakistan

## Abstract

*Adiantum capillus veneris* is a medicinally essential plant used for the treatment of diverse infectious diseases. The study of phytochemical and antimicrobial activities of the plant extracts against multidrug-resistant (MDR) bacteria and medically important fungi is of immense significance. Extracts from the leaves, stems, and roots of *Adiantum capillus veneris* were extracted with water, methanol, ethanol, ethyl acetate, and hexane and screened for their antimicrobial activity against ten MDR bacterial strains and five fungal strains isolated from clinical and water samples. Ash, moisture, and extractive values were determined according to standard protocols. FTIR (Fourier transform infrared Spectroscopy) studies were performed on different phytochemicals isolated from the extracts of *Adiantum capillus Veneris*. Phytochemical analysis showed the presence of flavonoids, alkaloids, tannins, saponins, cardiac glycosides, terpenoids, steroids, and reducing sugars. Water, methanol, and ethanol extracts of leaves, stems, and roots showed significant antibacterial and antifungal activities against most of the MDR bacterial and fungal strains. This study concluded that extracts of *Adiantum capillus veneris* have valuable phytochemicals and significant activities against most of the MDR bacterial strains and medically important fungal strains.

## 1. Introduction

Among foremost health problems, infectious diseases account for 41% of the global disease burden along with noninfectious diseases (43%) and injuries (16%) [1]. The main reasons of these infectious diseases are the natural development of bacterial resistance to various antibiotics [2, 3]. The development of multidrug-resistant (MDR) bacteria takes place because of the accumulation of different antibiotic resistance mechanisms inside the same strain [2, 4]. Although, in previous decades, the pharmacological companies have produced a number of new antibiotics, but even then drug resistance has increased [5]. This situation has forced the attention of researchers towards herbal products, in search of development of better-quality drugs with improved antibacterial, antifungal, and antiviral activities [6, 7].

According to world Health Organization (WHO), 80% of the World's population is dependent on the traditional medicine [8]. Herbal plants are rich sources of safe and effective medicines [9] and are used throughout the history of human beings either in the form of plant extracts or pure compounds against various infectious diseases [10]. For the treatment of infectious diseases, different medicinal plants have been mentioned by many phytotherapy manuals because of their reduced toxicity, uncomplicated availability, and fewer side effects [11]. Various studies have been conducted worldwide to describe the antimicrobial activities of different plant extracts [12–18]. Numerous plants have been investigated for treatment of urinary tract infections, gastrointestinal disorders, and respiratory and cutaneous diseases [19].

*Adiantum capillus veneris *is a common fern found in pak-indian subcontinent, Mexico, western Himalaya, warmer parts of America, and other tropical and subtropical regions of the world [20, 21]. It is used as expectorant, emmenagogue, astringent, demulcent, antitussive, febrifuge, diuretic and catarrhal affections [22]. Different extracts obtained from *Adiantum* had shown potential antibacterial activities against *Staphylococcus aureus*,* Streptococcus pyogenes, Klebsiella pneumoniae, Escherichia coli,* and antifungal activity against *Candida albicans* [8].

For few decades, phytochemicals (secondary plant metabolites), with unidentified pharmacological activities, have been comprehensively investigated as a source of medicinal agents [23]. Thus, it is expected that phytochemicals with sufficient antibacterial efficacy will be used for the cure of bacterial infections [10]. Many phytochemicals have been found in *Adiantum capillus veneris *like oleananes, phenylpropanoids, flavonoids, triterpenoids, carotenoids, carbohydrates, and alicyclics [24].

The present work was therefore designed to investigate the phytochemical, antibacterial, and antifungal activities of methanol, ethanol, water, ethyl acetate, and hexane extracts of leaves, stems, and roots of *Adiantum capillus veneris *against MDR bacterial strains isolated from community acquired and nosocomial infections and medicinally important fungi.

## 2. Materials and Methods

### 2.1. Plant Material Collection and Extraction

*Adiantum capillus veneris *was collected from different areas of Swat and Peshawar and then identified by the Department of Botany, University of Peshawar. For the collection of different extracts, the leaves, stems, and roots were separately shadow dried by the same method of Shalini and Sampathkumar [25]. The leaves, stems, and roots were separately ground to homogenous powder. 100 g of each powder, that is, leaves, stems, and roots was soaked in 1 liter of each distilled water, methanol, ethanol, ethyl acetate and hexane for 24 h at 25°C and then filtered through Whatman No. 1 filter paper. According to previously described methods, the filtrates were collected in separate flasks and the same process was repeated three times [26]. The filtrates, that is, crude extracts obtained were concentrated in rotary evaporator. For the isolation of pure extracts, the isolated crude extracts were resuspended in a minimum required volume of corresponding solvents and placed on the water bath (60°C) for the evaporation of extra solvents. These extracts were then preserved in separate containers for further experimentations at 5°C, according to previous methods [27].

### 2.2. Ash, Moisture, Extractive Values, Phytochemical Screening, and FTIR Study of Plant Extracts

Ash value of whole plant was found out by the method of Premnath et al. [28]. Moisture value of whole plant was determined by the same method as of Ashutosh et al. [29]. Extractive values of all the fifteen extracts of leaves, stems, and roots were carried out separately by the method described by Singh et al. [30]. Different types of phytochemical tests were performed for the presence of alkaloids, tannins, saponins, flavonoids, steroids, terpenoids, glycosides, and reducing sugars [31–33]. The FTIR (Fourier transform infrared Spectroscopy) model used was IR Pretige-21 (Shimadzu, Japan) with IR Solutions software [34]. FTIR spectroscopy was carried out for all the extracts in dried form by the method used by Meenambal et al. [35].

### 2.3. Collection and Identification of Bacterial Cultures

The bacterial samples were obtained from the laboratories of Lady Reading Hospital, Peshawar, and Pakistan council of scientific and industrial research (PCSIR), Peshawar. Bacterial species, that is, *Citrobacter freundii, Escherichia coli,* and *Providencia *species were isolated from urine samples, *Klebsiella pneumoniae, Proteus vulgaris, Salmonella typhi, Shigella,* and *Vibrio cholerae* from water sample while *Pseudomonas aeruginosa* and *Staphylococcus aureus *were isolated from pus samples. The isolated bacterial species were subcultured on selective and differential media, for example, CLED agar and MacConkey, and were identified through their specific characteristics, that is, morphological, staining, and biochemical, according to previously described methods [36].

### 2.4. Collection and Identification of Fungal Cultures

The fungal samples, that is, *Candida albicans*, *Pythium*, *Aspergillus flavis*, *Aspergillus niger,* and *Trichoderma*, were obtained from the microbiology laboratory of Abasyn University Peshawar. The collected fungal species were subcultured on potato dextrose agar (PDA) and were confirmed by staining and morphological characteristics according to the standard method [37].

### 2.5. Assessment of Drug Resistance Pattern of the Test-Bacterial Strains

Disk diffusion method was used for measurement of the antimicrobial activity on Muller Hinton agar. The sensitivity of fourteen antibiotics was tested against the previously mentioned ten bacterial strains (Table 3) and the process was repeated for three times. All the plates were incubated for 24 h at 37°C [38].

### 2.6. Evaluation of Antimicrobial Activity of Extracts

For the assessment of antimicrobial activities of all the fifteen extracts of *Adiantum capillus veneris*, the well diffusion method of Janovska et al. [39] was followed with some modifications. One mg of plant extract was dissolved in 1 mL of DMSO (dimethyl sulfoxide). Preautoclaved Muller Hinton agar plates were inoculated with a 10^−5^ dilution of bacterial cultures with sterile cotton swabs, for uniform growth. To test the activity of plant extracts, sterile cork borer was used to bore wells in the agar. 60 *μ*L of each extract, that is, LW (leaves water), LM (leaves methanol), LE (leaves ethanol), LEA (leaves ethyl acetate), LH (leaves hexane), SW (stem water), SM (stem methanol), SE (stem ethanol), SEA (stem ethyl acetate), SH (stem hexane), RW (root water), RM (root methanol), RE (root ethanol), REA (root ethyl acetate), and RH (root hexane), was introduced through micropipette aseptically into distinctively marked wells in the agar plates. All the plates were incubated for 24 h at 37°C and the process was repeated thrice.

### 2.7. Antifungal Activity of Plant Extracts

Well diffusion method of Mbaveng et al. [40] was used for the evaluation of antifungal activities of plant extracts. Preautoclaved PDA plates were inoculated with dilution of fungal cultures. 60 *μ*L of each extract, that is, SWE, SME, SEE, SEAE, SHE, LWE, LME, LEE, LEAE, LHE, RWE, RME, REE, REAE, and RHE was introduced through micropipette aseptically into distinctively marked wells in the agar plates. All the plates were incubated for 72 h at 37°C and the process was repeated in triplicate.

## 3. Results

### 3.1. Ash, Moisture, and Extractive Value

The ash value of the whole plant was 7.81% and moisture value was 10% while extractive values were separately calculated for all the 15 extracts. LM extract had a greater percentage of extractive value (35%) followed by REA (23.6%), SM (20%), LE (20%), RE (18%), RW (17.72%), SE (16.2%), RM (16%), SEA (12%), LW (12%), LEA (10.7%), LH (8%), RH (4.32%), SW (4%), and SH (2.75%) (Table 1).

### 3.2. Phytochemical Screening

It is evident from Table 2 that many phytochemicals were present in *Adiantum capillus veneris*.

### 3.3. FTIR Spectroscopy

**Figure 1:**
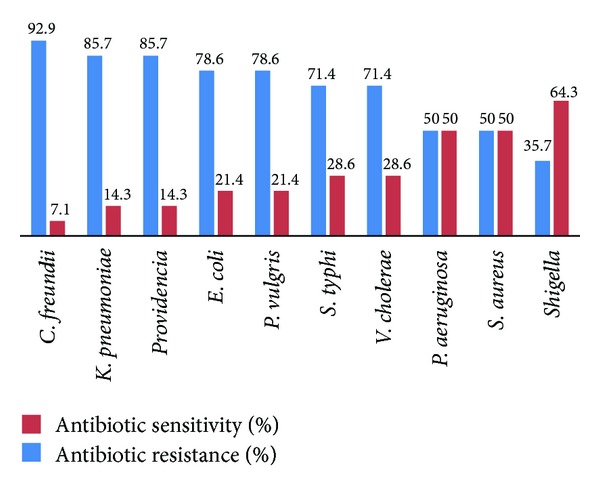
Percentage of antibiotic resistance and sensitivity of MDR bacterial strains.

FTIR spectroscopy was used for the compound identification and run under IR region between the ranges of 400 and 4000 cm^−1^. The peaks (see Figures 1 to 15 in Supplementary Material available online at http://dx.doi.org/10.1155/2014/269793) showed that the plant has compounds such as aldehyde, amides, alcohol, carboxylic acid, ketone and ethers, and so forth.

### 3.4. Drug Resistance Pattern of the Test-Bacterial Strains

The MDR bacterial strains were tested for antibiotic sensitivity against 14 frequently used antibiotics. Most of the tested bacterial strains were found to be resistant to the used antibiotics.* Citrobacter freundii* was the most resistant strain (92.8%) that showed relatively low sensitivity only to tetracycline (TET) (10 mm), among all the tested organisms. Second most resistant strain (85.7%) was *Klebsiella pneumoniae *which showed sensitivity only to gentamicin (GEN) (15 mm) and cefoperazone-sulbactam, (CZS) (15 mm) followed by *Providencia *(85.7%), which showed sensitivity to cefotaxime (CTX) (22 mm) and ceftriaxone (CRO) (18 mm). *Proteus vulgaris *and *Escherichia coli *were 78.6% resistant while *vibrio cholera* and* Salmonella typhi* were 71.4% resistant to all tested antibiotics. *Pseudomonas aeruginosa* and *Staphylococcus aureus* were found 50% resistant while *Shigella* was 35.8% resistant against all 14 test- antibiotics (Table 3).

### 3.5. Assessment of Antibacterial Activity of Plant Extracts

The leaves, stems and root extracts of *Adiantum capillus veneris *were tested against ten MDR bacterial strains. 60 *μ*L (1 mg/1 mL) of each extract was used for antimicrobial activity estimation through well diffusion method. LM, LE, LW, SM, SE, SW, RM, RE, and RW extracts of *Adiantum capillus veneris *showed significant antibacterial activity against all the test bacterial strains (Table 4).

The results were recorded after a 24-hour incubation, according to the ZI of each antibiotic for all tested bacterial strains.

### 3.6. Assessment of Antifungal Activity of Plant Extracts

Water, methanol, and ethanol extracts of leaves, stems, and roots of *Adiantum capillus veneris* showed maximum ZI against tested fungal strains while hexane extract of leaves, stems and, roots has shown no activity. LM extract has shown highest zone against *Candida albicans *(30 ± 1.00 mm), *Aspergillus flavis *(30 ± 1.00 mm), *Aspergillus niger *(30 ± 1.00 mm), *Pythium *(28 ± 1.00 mm), and *Trichoderma *(28 ± 1.00 mm). Similarly, LW, LE, LE, LEA, SW, SM, SEA, RW, RM, RE, and REA were also very active against most the test-fungal strains as evident from Table 5.

## 4. Discussion

The attention of researchers has been deviated by the increasing emergence of antibiotic resistance towards the medicinal plants in search of new, less toxic, and useful drugs. Plants are the reservoirs of valuable phytochemicals. Many plants have been investigated worldwide for their antimicrobial and phytochemical activities. Therefore, this study has been carried out to evaluate the phytochemical and antimicrobial activities of water, methanol, ethanol, ethyl acetate, and hexane extracts of leaves, stems, and roots of *Adiantum capillus veneris*.

Ash, moisture, and extractive values of all fifteen extracts of *Adiantum capillus veneris* were determined. Except for the ash value of whole plant which is in accordance with the study of Ahmad et al. [22], the moisture and extractive values reported in our study have not been investigated before, to the best of our knowledge.

The result of phytochemical screening of all extracts of leaves, stems, and roots of *Adiantum capillus veneris* showed the presence of alkaloids, flavonoides, tannins, saponins, terpenoids, steroids, glycosides, and reducing sugars (Table 2) which is in line with many other studies conducted worldwide [25, 41, 42]. FTIR results of our study have showed the presence of many new compounds, that is, aldehyde, amides, alcohol, carboxylic acid, ketone, and ethers (Figures 1–15, supplementary data), most of which are not reported previously.

In the present study, 10 bacterial strains were used which were MDR to most of the given antibiotics (Table 3). Our results showed that *Citrobacter freundii *was the most resistant strain (92.8%) among all the tested bacterial strains. Our findings are in line with the studies conducted in other areas of Pakistan where 100% MDR *Citrobacter* has been reported [43]. Additionally, 92.8% MDR *Citrobacter* seen in the present study is also observed in Ethiopia (100% MDR) [44] and Nepal (86.95%) [45]. Similarly, 85.7% MDR *Klebsiella pneumoniae *found in this study is almost in agreement with 81.8% MDR investigated locally [46] in early 2013. We investigated 85.7% MDR *Providencia*, almost similar to the study of Tumbarello et al. (75%) [47]. We have also investigated that *Escherichia coli*, *P. vulgaris, Salmonella typhi*, *V. cholera*, *Staphylococcus aureus, Pseudomonas aeruginosa,* and *Shigella* are rather more MDR (Figure 1) than what was found in other regions of the world, as evident from various studies [48–50] on these bacterial strains.

Numerous studies on *Adiantum capillus veneris *showed its potency against MDR bacterial strains. For example, *Escherichia coli, Staphylococcus aureus,* and *Klebsiella Pneumoniae *were sensitive to LW, LM, SW, and SM extracts of *Adiantum capillus veneris* in our study which proved to be almost in accordance with the findings of Mahboubi et al. [51] and kumar and Nagarajan [8] from Iran and India, respectively. We have found out that most of the extracts of *Adiantum capillus veneris *were very effective against the MDR bacterial strains as compared to other studies [52, 53] which might be due to the variation in procedures, geographical conditions, and so forth. In comparison to the antibiotics used, the plants extracts were very active against the test bacterial strains, which is evident from the comparison of Tables 3 and 4. Likewise, as compared to other studies [22, 54], all extracts except hexane used in our studies were far more effective against test-fungal strains.

The present study confirms that fractions of *Adiantum capillus veneris *have significant antibacterial and antifungal activity along with valuable phytochemicals. Different fractions have different antibacterial and antifungal activities against MDR bacterial and fungal strains. It is recommended that further research should be conducted for more effective outcomes.

## Supplementary Material

FTIR spectroscopy was used for the compound identification and run under infra red (IR) region between the ranges of 400-4000 cm^−1^. The phytochemical constituents were confirmed by FTIR. The peaks showed that the plant have compounds such as Aldehyde, ketone, alcohol, carboxylic acid, amides and ethers etc. (Figures 1 to 15)

## Figures and Tables

**Table 1:** Ash, moisture, and extractive values of fifteen extracts of *Adiantum capillus veneris*.

Plant part	Solvent	Extractive value (%)	Moisture value (%)	Ash value of whole plant (%)
Leaves	Water	12	10	7.81
	Methanol	35		
	Ethanol	20		
	E. acetate	10.7		
	Hexane	8		
Stems	Water	4		
	Methanol	20		
	Ethanol	16.2		
	E. acetate	12		
	Hexane	2.75		
Roots	Water	17.72		
	Methanol	16		
	Ethanol	18		
	E. acetate	23.6		
	Hexane	4.32		

**Table 2:** Phytochemicals detected in different extracts of *Adiantum capillus veneris*.

Plant part	Solvent	Alkaloids	Flavonoids	Tannins	Saponins	Terpenoids	Steroids	Glycosides	Reducing sugar
Leaves	Water	+	+	+	+	+	+	+	+
	Methanol	+	+	+	+	+	+	+	+
	Ethanol	+	+	+	+	+	+	−	−
	E. acetate	+	+	+	+	−	+	−	−
	Hexane	+	+	+	+	+	+	−	−

Stems	Water	+	+	+	+	+	+	+	+
	Methanol	+	+	+	+	+	+	+	+
	Ethanol	+	+	+	+	+	+	−	−
	E. acetate	+	+	+	+	+	+	−	−
	Hexane	+	+	−	+	−	+	−	−

Roots	Water	+	+	+	+	+	+	+	+
	Methanol	+	+	+	+	+	+	+	+
	Ethanol	+	+	+	+	+	+	−	−
	E. acetate	+	+	+	+	+	+	−	−
	Hexane	+	+	−	+	+	+	−	−

**Table 3:** Drug resistance pattern of the test-bacterial strains.

S. no.	Microorganisms	Antibiotic discs with ZI (mm) representing sensitivity, while (—) representing resistance													
		1 AMP	2 AMX	3 CF	4 CPH	5 CIP	6 CTX	7 CRO	8 CZS	9 GEN	10 MXF	11 NA	12 NOR	13 TET	14 TS
1	*E. coli *	—	—	—	—	—	—	—	—	18	10	—	—	20	—
2	*C. freundii *	—	—	—	—	—	—	—	—	—	—	—	—	10	—
3	*K. pneumonia *	—	—	—	—	—	—	—	15	15	—	—	—	—	—
4	*S. typhi *	—	—	—	—	—	15	—	—	—	10	9	—	11	—
5	*Shigella *	20	—	—	—	28	30	—	20	12	19	19	29	11	—
6	* P. vulgaris *	—	—	—	—	—	—	—	20	13	—	—	—	10	—
7	*Providencia *	—	—	—	—	—	22	18	—	—	—	—	—	—	—
8	*P. aeruginosa *	—	—	—	—	30	16	—	30	14	28	—	30	—	12
9	*Staph. Aureus *	—	—	—	—	30	25	—	25	18	25	—	30	12	—
10	*V. cholerae *	—	—	—	—	—	21	21	—	12	—	21	—	—	—

AMX: amoxicillin, AMP: ampicillin, CF: cefaclor, CIP: ciprofloxacin, CPH: cephradine, CTX: cefotaxime, CZS: cefoperazone-sulbactam, CRO: ceftriaxone, GEN: gentamicin, MXF: moxifloxacin, NA: naladixic acid, TET: tetracycline, NOR: norfloxacin, TS: trimethoprim-sulfamethoxazole, ZI: zone of inhibition.

**Table 4:** Antibacterial activity of fifteen extracts of *Adiantum capillus veneris* against various MDR bacterial strains.

Plant part	Solvent	*E. coli *	*Pseudomonas *	*Citrobacter *	*Klebsiella *	*Proteus *	*Vibrio *	*Shigella *	*Salmonella *	*S. aureus *	*Providencia *
Leaves	Water	20	25	20	25	25	25	20	22	20	20
	Methanol	18	15	22	30	25	30	30	25	28	30
	Ethanol	16	20	20	25	25	30	25	20	22	25
	E. acetate	15	15	0	10	0	20	15	20	0	15
	Hexane	15	15	0	0	0	0	12	0	0	0

Stems	Water	20	10	10	20	15	10	15	20	12	15
	Methanol	30	20	20	25	20	20	18	25	18	20
	Ethanol	30	25	18	25	25	20	20	30	18	20
	E. acetate	20	20	12	0	0	0	0	12	10	0
	Hexane	20	15	0	0	0	0	0	10	0	0

Roots	Water	25	22	25	20	20	30	25	20	18	10
	Methanol	25	22	20	18	15	20	12	15	15	15
	Ethanol	25	20	20	16	20	20	20	15	20	15
	E. acetate	20	25	14	20	15	18	15	10	14	10
	Hexane	0	0	0	0	0	0	0	0	0	0

Extracts with zone of inhibition (ZI) representing sensitivity in millimeter (mm).

**Table 5:** Antifungal activity of *Adiantum capillus veneris* extracts.

Plant part	Solvent	*Candida albicans *	*Trichoderma *	*Pythium *	*Aspergillus flavis *	*Aspergillus niger *
Leaves	Water	20	22	24	25	25
	Methanol	30	28	28	30	30
	Ethanol	25	25	25	28	28
	E. acetate	15	14	20	20	16
	Hexane	0	0	0	0	0

Stems	Water	18	15	20	18	20
	Methanol	20	18	22	20	18
	Ethanol	20	16	20	20	18
	E. acetate	0	10	12	10	12
	Hexane	0	0	0	0	0

Roots	Water	25	22	25	20	22
	Methanol	20	20	20	22	25
	Ethanol	20	18	18	25	20
	E. acetate	0	10	14	12	10
	Hexane	0	0	0	0	0

Extracts with zone of inhibition (ZI) representing sensitivity in millimeter (mm).
