# Supplementary material for: In Vitro Phytochemical, Antibacterial, and Antifungal Activities of Leaf, Stem, and Root Extracts of Adiantum capillus veneris
Source: ScientificWorldJournal. 2014 Jan 28;2014:269793. doi: 10.1155/2014/269793 (PMC3925560; doi:10.1155/2014/269793)
Supplement: Supplementary file 1 — FTIR spectroscopy was used for the compound identification and run under infra red (IR) region between the ranges of 400-4000 cm−1. The phytochemical constituents were confirmed by FTIR. The peaks showed that the plant have compounds such as Aldehyde, ketone, alcohol, carboxylic acid, amides and ethers etc. (Figures 1 to 15) [file 269793.f1.doc]

FIGURE 1: FTIR spectroscopy of leaves water extract of *Adiantum capillus veneris*

FIGURE 2: FTIR spectroscopy of leaves methanol extract of *Adiantum capillus veneris*

FIGURE 3: FTIR spectroscopy of leaves ethanol extract of *Adiantum capillus veneris*

FIGURE 4: FTIR spectroscopy of leaves ethyl acetate extract of *Adiantum capillus veneris*

FIGURE 5: FTIR spectroscopy of leaves hexane extract of *Adiantum capillus veneris*

FIGURE 6: FTIR spectroscopy of stem water extract of *Adiantum capillus veneris*

FIGURE 7: FTIR spectroscopy of stem methanol extract of *Adiantum capillus veneris*

FIGURE 8: FTIR spectroscopy of stem ethanol extract of *Adiantum capillus veneris*

FIGURE 9: FTIR spectroscopy of stem ethyl acetate extract of *Adiantum capillus veneris*

FIGURE 10: FTIR spectroscopy of stem hexane extract of *Adiantum capillus veneris*

FIGURE 11: FTIR spectroscopy of root water extract of *Adiantum capillus veneris*

FIGURE 12: FTIR spectroscopy of root methanol extract of *Adiantum capillus veneris*

FIGURE 13: FTIR spectroscopy of root ethanol extract of *Adiantum capillus veneris*

FIGURE 14: FTIR spectroscopy of root ethyl acetate extract of *Adiantum capillus veneris*

FIGURE 15: FTIR spectroscopy of root hexane extract of *Adiantum capillus veneris*
